# Sweet Round, Spicy Sharp: How Chinese Typeface Features Trick the Tongue into Tasting Five Flavors

**DOI:** 10.3390/bs16060913

**Published:** 2026-06-03

**Authors:** Chunfa Sha, Yunxiao Zhang, Qi Zhong, Haojing Guo, Rong Han

**Affiliations:** 1 Department of Industrial Design, Jiangsu University, Zhenjiang 212000, China; 2 Department of Design, North Carolina State University, Raleigh, NC 27606, USA

**Keywords:** Chinese typography, taste expectancy, cross-modal correspondence, food packaging

## Abstract

Cross-modal correspondence, a cross-sensory cognitive phenomenon, reveals the link between visual forms and taste experiences. Although research on Western alphabetic typography is extensive, the Chinese character system has received less attention. This study systematically examines Chinese typeface categories (Song, Hei, Yuan, and Calligraphic) and stroke thickness (bold, medium, thin) in a real packaging context. The effects on the expectancies of the five flavors—sour, sweet, bitter, spicy, and salty—were measured. The results showed a significant interaction effect between typeface category and stroke thickness for all five flavor dimensions. The rounded Yuan typeface with thin strokes tended toward sweetness. Bold Song typefaces tend toward bitterness or spiciness, while bold Hei typefaces tend toward saltiness. The Calligraphic typeface, with its complex visual features, is strongly associated with bitterness and spiciness only when paired with bold strokes. Sourness shows a reverse modulation pattern. These findings reveal that the visual features of Chinese typefaces shape gustatory crossmodal correspondences through interactive modulation rather than simple linear addition. This confirms the common cross-modal perceptual pattern of ‘curvature-sweetness, angularity-non-sweetness’ across Chinese and English writing systems while also reflecting the culturally specific patterns driven by Chinese typographic features (such as the bifeng structure at stroke terminals) and the unique phenomenon of cross-modal correspondence with spiciness. This provides an empirically grounded and culturally adaptive reference for the marketing application of Chinese typefaces in packaging contexts.

## 1. Introduction

### 1.1. Crossmodal Correspondence: From Shape to Typeface–Universality and Application

Crossmodal correspondence refers to the cognitive phenomenon in which systematic matching relationships exist between features across different sensory channels. It has become a frontier topic in contemporary cognitive science research (Spence, 2011). Unlike clinical synesthesia, crossmodal correspondence reflects environmental statistical regularities acquired and automated through daily experience. This phenomenon essentially represents the human sensory system’s adaptive coding of the world’s regularities. These correspondences operate largely outside of awareness. Yet, they significantly and robustly influence human perception, judgment, and behavior. A large body of empirical research has fully confirmed the universality and diversity of crossmodal correspondences. For example, in temperature-vision crossmodal correspondences, systematic associations exist between colour hue and temperature perception (Spence, 2020). In the sound–shape dimension, high-frequency tones are often perceived as bright, sharp visual forms. In contrast, low-frequency tones are associated with heavy, rounded shapes (Loconsole et al., 2023; Sciortino & Kayser, 2023). Colour and shape significantly influence the perception of sweetness, sourness, and aroma of foods (Chen et al., 2021; Motoki & Velasco, 2021; M. Wang & Li, 2022). These findings collectively reveal the authenticity and cross-domain consistency of crossmodal correspondence as a universal cognitive mechanism.

Among the many studies on crossmodal correspondence, the mapping between shape and taste has received particularly robust empirical support. This topic has also developed a relatively mature theoretical framework. Spence (2011) proposed a four-category framework for crossmodal correspondence mechanisms: statistical correspondence (statistical learning based on environmental regularities), affective correspondence (mediated by affective associations), semantic correspondence (rooted in linguistic and conceptual links), and structural correspondence (reflecting isomorphic mapping of stimulus dimensions). The statistical learning hypothesis suggests that individuals acquire consistent associations of sensory features through repeated co-occurrence in the natural environment. During long dietary experience, smooth, rounded textures often co-appear with sweet foods (e.g., ripe fruits). Sharp, angular shapes often accompany sour or bitter substances (e.g., unripe fruits, medicinal plants). Empirical research has confirmed this: curved and rounded features positively predict sweetness perception (Velasco et al., 2014; Motoki et al., 2019), whereas angular and sharp features are associated with sour or bitter taste expectancy (Velasco et al., 2015a, 2016a, 2016b). The affective mediation hypothesis also suggests that rounded, curved shapes elicit more positive affective responses than sharp, angular shapes. Since sweetness naturally carries positive valence (Drewnowski, 1997), this partly explains the round–sweet correspondence (Motoki et al., 2019; Salgado-Montejo et al., 2015). This concept has also been applied to the design of food shapes (Spence et al., 2013).

In a cross-cultural study covering 25 languages and 10 writing systems, Cwiek et al. (2022) found that the bouba/kiki effect was robust across cultures and writing systems. This suggests that shape–taste crossmodal phenomena may have an innate basis. The same phenomenon exists in the field of typeface design. Velasco et al. (2015b) systematically demonstrated stable correspondences between typeface curvature and basic taste words. Rounded typefaces are more readily matched with sweetness. Angular typefaces pair well with salty, sour, and bitter flavors. The psychological basis lies in the higher processing fluency and positive affective valence of rounded typefaces. Typefaces can simultaneously convey taste quality, intensity, and affective information (Velasco et al., 2018).

### 1.2. Deepening of Western Typeface–Taste Crossmodal Correspondence Research

The above studies indicate that the shape–taste crossmodal phenomenon in Western typefaces is generalizable, and continued attention has been given to the effects and interactions of other visual features as well as the ecological validity in real consumption contexts.

Stroke thickness or weight. Spence (2011) pointed out that intensity matching in crossmodal correspondences (e.g., brightness and loudness, visual weight and auditory intensity) may have a physical correspondence property independent of quality pathways. Thicker strokes, due to their greater visual weight, may imply intense flavours such as bitterness and saltiness, whereas thin strokes imply mild flavours. When bold strokes in angular typefaces are combined with the sharpness of angularity, an enhancing effect on intense tastes such as bitterness occurs (Velasco et al., 2018), suggesting that designers should synergistically configure curvature and weight.

Interaction effects. Typeface curvature and weight do not operate independently; rather, they jointly shape taste expectancy through synergistic interactions. Sunaga et al. (2025) found that the connotative meanings of instrumental timbre (e.g., bright, smooth, heavy) and the connotative consistency between visual design features significantly influence consumer evaluations and choice behavior. This crossmodal connotative congruency effect suggests that when the visual features of a typeface align with the connotative dimension of the taste image it conveys, consumers experience higher processing fluency and a stronger sense of sensory correctness, leading to more positive evaluations. Lee and Spence (2023) systematically integrated the interactions of visual features such as colour, shape curvature, and texture, constructing a theoretical framework to predict the overall effect of visual feature combinations, emphasizing that the taste expectations generated by such combinations may deviate from the simple summation of individual features and can even give rise to entirely new semantic meanings. Piqueras-Fiszman and Spence (2011) and Piqueras-Fiszman et al. (2012), using implicit association tests to study packaging colour–taste correspondences, found that brand familiarity and learning experience played significant moderating roles. This finding suggests that typeface–taste correspondences may be influenced by both innate predispositions and acquired learning, and that examining the interaction between typefaces and brand logos, cultural symbols, etc., in real packaging contexts is an unavoidable direction for future research.

Real-world validation. Extending typeface–taste correspondences to real consumption contexts is a crucial step for validating ecological validity. de Sousa et al. (2020), using coffee packaging as a context, found that angular typefaces significantly increased consumers’ expected and perceived coffee acidity, whereas rounded typefaces showed no significant association with sweetness (possibly because coffee is predominantly bitter and sour), indicating that typeface effects are moderated by product category and are not universal or constant. Otterbring et al. (2022) found that angular typefaces were consistently associated with sourness, but the typeface effect was significantly attenuated among young consumer groups, suggesting that activation strength may vary with generational characteristics.

Although typeface–taste crossmodal correspondences have a certain degree of universality, they are modulated by cultural experience. Wan et al. (2014) documented cultural differences in crossmodal taste associations among participants from China, India, Malaysia, and the United States, demonstrating that cultural familiarity with specific food–visual pairings can enhance or modify crossmodal mappings. This finding suggests that directly generalising conclusions from Western typeface research to the Chinese writing system requires caution, because Chinese characters are not only logographic but also carry unique calligraphic traditions and visual aesthetics.

### 1.3. Research on Typeface–Taste Correspondences in the Chinese Context

Compared with the abundant research on Western typefaces, research on the Chinese character system remains relatively limited. The most directly related study comes from W. Wang (2017), who systematically tested crossmodal correspondences between the shape and brightness of Chinese characters and taste perception among Chinese and Malaysian participants. Using a two-survey design, Wang manipulated the shape (angular vs. rounded) and brightness (low, medium, high) of Chinese characters, and measured participants’ taste intensity and quality expectations. The results showed that angular Chinese characters were consistently associated with intense tastes (sour, bitter, salty), whereas rounded Chinese characters were associated with mild tastes and sweetness; characters with low brightness (dark colour) were rated as having a more intense taste, while those with high brightness (light colour) were linked to milder taste expectations. Crucially, the shape–brightness consistency analysis revealed that the combination of angular shapes with low brightness maximised intense taste expectations, while the combination of rounded shapes with high brightness maximised mild taste expectations. This suggests that shape quality and visual weight operate through partially independent pathways, producing synergistic effects.

In addition, research on synaesthesia in Chinese has revealed a language-internal mechanism that may reinforce cross-sensory correspondences among Chinese speakers. For example, Zhao et al.’s (2018) corpus analysis of gustatory adjectives showed that the intensity adjectives ‘浓’ (strong or dense) and ‘淡’ (light or pale) apply to both visual intensity—such as ‘浓绿’ (dark green; depth or saturation) and ‘淡蓝’ (light blue; paler hue)—and auditory intensity—such as ‘浓重的口音’ (strong accent; intensifies strength). They also found that quality-specific taste adjectives like ‘甜’ (sweet) often appear in visual and auditory contexts, such as ‘甜美的笑容’ (pleasant, sweet smile) and ‘甜美的声音’ (sweet, melodious voice). These findings support Spence’s proposal that repeated exposure to these linguistic patterns enables Chinese speakers to learn that intensity is a shared dimension of taste and vision. Finally, Zhao et al. (2017) showed that taste often serves as the source domain in Chinese synaesthetic transfers, suggesting it underlies Chinese cross-modal cognition.

### 1.4. Differences in Typeface–Taste Research Between Chinese and English Contexts

#### 1.4.1. Differences in the Basic Flavor Framework

Western food science typically recognises five basic tastes: sour, sweet, bitter, salty, and umami, whereas the traditional Chinese five-flavor theory centres on sour, sweet, bitter, spicy, and salty. This difference is deeply rooted in cultural traditions: the Yin-Yang and Five Elements theory in traditional Chinese philosophy laid the theoretical foundation for the five flavors, associating sour, bitter, salty, spicy, and sweet with the attributes of Metal, Wood, Water, Fire, and Earth, respectively, in the dimension of flavor. In traditional Chinese medicine (TCM), these five flavors also serve as key indicators of medicinal properties (T. Y. Huang et al., 2017). As ‘medicine and food share the same origin’, the five flavors are not only medicinal characteristics but are also deeply embedded in the daily diet, becoming central to the attributes of food ingredients and culture. As stated in the *Huainanzi·Yuan Dao Xun*: ‘味之和不过五，而五味之化不可胜尝也’ (The harmonies of taste are no more than five, yet the transformations of the five flavors are inexhaustible in tasting) (Xu, 2014). In the Chinese context, most scholars have studied sour, sweet, bitter, spicy, and salty as basic taste words, including Zhang (1999), Gou (2003), B. Z. Huang (2006), and Wertz (2007).

From a physiological perspective, spiciness is not a pure taste but a mixture of thermal sensation, pain, and basic taste qualities (He et al., 2023). However, spiciness has long been deeply rooted in Chinese culture and daily diet. In contrast, modern Western and Japanese food science have recognized umami as a basic taste, with Japanese researcher Kikunae Ikeda discovering it in 1908, long after the formal establishment of the Chinese five-flavor system. To maintain the cultural and historical consistency of the research framework, the present study deliberately focuses on the traditional five flavors. This cultural difference implies that investigating crossmodal correspondences, including spiciness, in the context of Chinese characters has both unique theoretical significance and indigenous cultural value.

#### 1.4.2. Differences in Visual Features of Typefaces

Bifeng is a distinctive visual feature of Chinese typefaces. Luo et al. (2025) described it as ‘stroke-specific terminals’, while the calligraphic tradition defines bifeng as the sharp, exposed tip traces produced at the beginning and end of a stroke due to the brush tip being revealed (Zhou, 2022).

Chinese bifeng is cross-scripturally parallel to serifs in Latin typefaces, both in form and function. Serif typefaces (e.g., Times New Roman) add small decorative strokes at the ends of strokes, thereby providing additional perceptual anchors to facilitate letter discrimination (Arditi & Cho, 2005), whereas sans-serif typefaces (e.g., Arial) omit these decorations (Bringhurst, 2004). The stroke-terminal features corresponding to Chinese bifeng have also been shown to be key diagnostic cues in Chinese character recognition (Luo et al., 2025). Li and Wang (2018) compared Song and Times New Roman typefaces across six aspects (number of strokes, stroke density, shape of stroke terminal angles, decoration of stroke terminal angles, etc.), pointing out that the shape and decoration of stroke terminals are important factors affecting typeface legibility, and confirmed that the geometric quantification of bifeng features is mapped onto perceptual and aesthetic evaluation. The presence or absence of bifeng forms a structural dichotomy in Chinese characters.

However, the forms of Chinese bifeng are much more complex. X. Wang et al. (2013) identified geometric information about Chinese character stroke terminals (e.g., sharpness, angle, curvature) as important bases for describing bifeng. They developed a method to calculate the stroke tip’s initial position using ‘sar(s)’, established a bifeng shape description model (multi-scale Fourier descriptor of curvature histogram, MFDCH), and described its visual style characteristics using stroke tip smoothness (SM). Yi et al. (2014) captured calligraphic style features in terminal segments, corner segments, and head segments, reflecting various styles with smooth contours and beautiful appearances. This means that bifeng is not merely a qualitative concept at the calligraphic or aesthetic level, but an objective geometric feature that can be detected, measured, and used in pattern recognition. But these studies have focused primarily on font recognition and have not conducted in-depth quantification of bifeng geometric features.

Rooted in the tradition of Chinese calligraphic aesthetics, the visual sharpness and range of morphological variation of bifeng are generally significantly greater than those of typical Western serifs, which may produce a stronger angularity effect in crossmodal correspondences. Therefore, directly applying conclusions from Western typeface research while ignoring the specific feature of bifeng in Chinese characters may lead to misjudgements of Chinese consumers’ flavour expectations. Given that the understanding of the relationship between bifeng geometric features and taste is still at an early exploratory stage, the present study first treats the presence or absence of bifeng as a binary variable to determine whether this feature modulates taste expectancy.

### 1.5. Research Approach and Hypotheses

Based on the above analysis, although Western research has established a basic framework linking typeface curvature to taste quality and typeface weight to taste intensity, and has revealed interaction effects among visual features, transplanting these conclusions directly to the Chinese context faces three research gaps that have not been sufficiently addressed. First, existing research lacks an examination of crossmodal correspondences grounded in the traditional Chinese five-flavor framework. Western research is based on sour, sweet, bitter, salty, and umami, whereas spiciness occupies a fundamental position in Chinese food culture; its mapping onto the visual features of Chinese characters has not yet been systematically tested. Second, the bifeng feature unique to Chinese characters has long been overlooked. Existing Chinese studies have manipulated only the macro-level curvature and brightness of typefaces, without including bifeng as an independent variable; consequently, it remains unclear to what extent the ‘angularity effect’ in Western research is driven by Chinese bifeng, let alone the specific association between bifeng and intense tastes such as spiciness. Third, no study has systematically investigated the interaction between typeface category and stroke thickness in Chinese typeface–taste correspondences. W. Wang (2017) examined the synergistic effect of shape and brightness but did not address stroke thickness, a key intensity cue; the possible non-linear synergistic or inhibitory relationship between typeface category (reflecting the presence/absence of bifeng, morphological differences, and overall curvature) and stroke thickness remains an unexplored area.

Unlike the main-effect testing approach used in Western research, the present study focuses on the interactive effects of typeface category and stroke thickness on the five Chinese flavors in a concrete packaging context. Accordingly, we hypothesize that in the Chinese writing system, different typeface categories and the visual weight represented by stroke thickness exhibit a non-linear synergistic or inhibitory relationship: typeface category moderates the direction and strength of the effect of stroke thickness, while the transmission of taste expectancy via stroke thickness also depends on the specific visual attributes of the typeface category. In other words, the two do not independently influence taste judgments; rather, they interact and jointly shape the final taste expectancy.

## 2. Exploratory Experiment

### 2.1. Methods

#### 2.1.1. Screening of Samples for the Exploratory Experiment

The exploratory experiment aimed to screen and verify the associative links between different Chinese typeface categories and the five flavors, thereby providing foundational materials for the formal experiment. Based on the font classification system and download frequency data from the Ziyou platform (https://www.hellofont.cn/), combined with the classification criteria for packaging typeface design outlined in relevant literature (Velasco et al., 2018; Xiong, 2004), 20 representative font samples were preliminarily selected and categorized into five major types: calligraphic fonts, classical-style fonts, modern fonts, creative fonts, and artistic fonts (Table 1).

**Figure 1:**
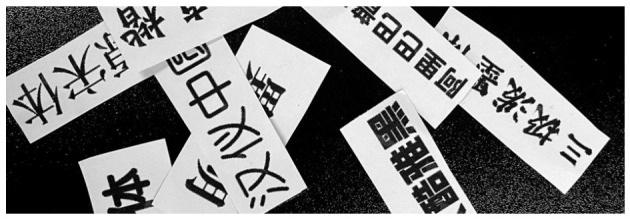
Physical test cards in the card sorting task.

We used the card sorting method to screen the samples and select representative typeface stimuli, thereby improving the feasibility of subsequent experiments (Martin & Hanington, 2013). First, we produced cards with the typeface displayed on one side and the reverse side left blank (Figure 1) to prevent participants from being influenced by prior knowledge that might interfere with their initial intuitive judgments. Second, five design researchers were invited to evaluate and screen the 20 preliminary samples based on visual features such as bifeng and stroke thickness. This method leveraged diverse perspectives from researchers to ensure the preliminary sample set was representative and comprehensive, thereby enhancing the accuracy and completeness of the research findings. Ultimately, four groups (comprising eight typefaces in total) were selected for the next stage of the experiment (Table 2). The specific steps of the card sorting procedure were as follows:

(1) Grouping and naming: Based on visual features, the five designers independently classified the 20 typeface cards into groups by placing visually similar ones together, and gave each group a name.
(2) Reason elaboration: Each designer provided a written explanation of the visual rationale for each self-created category and any associated sensory impressions.
(3) The researcher compiled results and analyzed major grouping trends, consensus features, and differing viewpoints.
(4) Sample selection: Based on the textual analysis and K-means clustering results, the researcher selected representative samples and described their characteristics.

Based on the oral reports, the researchers’ primary classification criteria included the presence or absence of bifeng, contrast in stroke thickness, and overall morphological features. Combined with K-means clustering, the 20 preliminary samples were divided into four categories: Category 1—Song typeface (with bifeng, pronounced stroke-thickness contrast, upright glyph shape); Category 2—Hei typeface (without bifeng, uniform stroke thickness, upright glyph shape); Category 3—Yuan typeface (without bifeng, uniform stroke thickness, rounded glyph shape); Category 4—Calligraphic typeface (strokes with a symbolic character, distinctive writing style). These four categories form an orthogonal structure along the two core dimensions of bifeng and stroke thickness, ensuring both systematicity and representativeness of the visual features.

**Figure 2:**
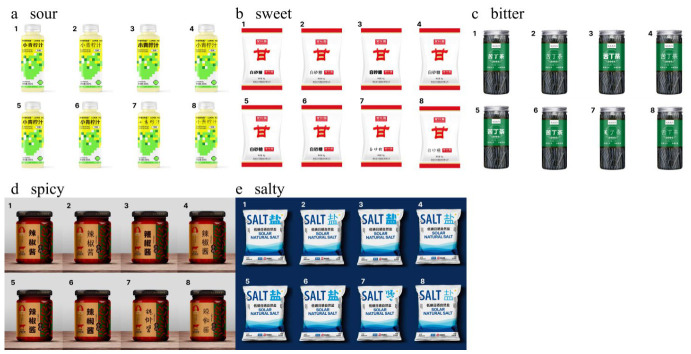
Stimuli used in the exploratory experiment: (**a**) sour taste stimuli, (**b**) sweet taste stimuli, (**c**) bitter taste stimuli, (**d**) spicy taste stimuli, (**e**) salty taste stimuli.

To ensure that the research focused on the crossmodal effects of typefaces in a packaging context, the eight selected typefaces were applied to common food packaging representing the five flavors. All products were chosen from the most common categories in the Chinese daily diet (lemon water, white sugar, Kuding tea, chili sauce, and table salt) to ensure that participants had highly certain and consistent taste expectations for each product, thereby establishing a comparable baseline within each product and allowing the modulatory effect of typefaces to be detected. In the experiment, visual elements such as patterns and colour schemes were kept identical across all packaging, with typeface as the only variable manipulated, to isolate the interference of other packaging design factors to the greatest extent and to test the effect of typeface features on taste expectancy (Figure 2).

#### 2.1.2. Participants

Participants in the study were required to have a Chinese cultural background to ensure the experimental stimuli could be effectively activated. Designers who had participated in the preliminary card sorting were excluded from this experiment, thereby ensuring that all participants were exposed to the experimental stimuli for the first time. As shown in Table 3, 80 participants were recruited (*M_age_* = 35.28 ± 14.05; 51.25% female; sample size similar to that of Velasco et al. (2018), with the majority aged 18–44 years (61.25%). This study used an anonymous questionnaire survey with minimal risk to participants. Voluntary completion and return of the questionnaire were taken to imply informed consent. Therefore, ethical approval was exempted, and the requirement for written informed consent was waived.

#### 2.1.3. Procedure

Data were collected online via the Wenjuanxing platform (https://www.wjx.cn/). The platform used a forced-response feature. This ensured that all data were collected completely and that no questions were omitted. Participants read an informed consent statement and confirmed their agreement before entering the experimental task. They had the right to withdraw from the experiment at any time. If they withdrew, their data were excluded from the final analysis.

The experiment consisted of five sets of stimulus materials, corresponding to the five flavors: sour, sweet, bitter, spicy, and salty. Each set was composed of eight typeface images (Figure 2). The images in each set were presented in random order to eliminate order effects. A 30 s adaptation period was provided before each set. During adaptation, participants observed all images in that set to familiarize themselves with the materials. After the adaptation period, participants answered the following question for each of the eight images in the current set: ‘Based on your first impression, please select the image(s) that most strongly evoke the following taste perception (multiple selections allowed, up to three).’ Participants completed the five sets of tasks independently. Each set was conducted separately, and no feedback was provided between sets.

Throughout the experiment, the experimenter did not interfere with participants’ judgments and was responsible only for recording evaluation results and participants’ verbal feedback (e.g., intuitive descriptions of typeface features). All data were collected anonymously and used solely for academic research. After the experiment, participants received a cash red envelope as a token of appreciation.

### 2.2. Results Analysis

**Figure 3:**
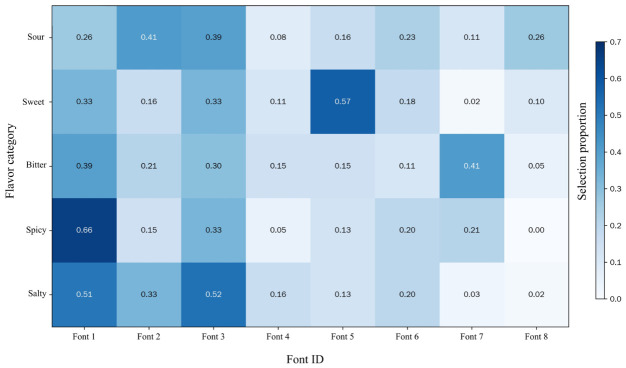
Heatmap of the distribution of selection proportions for different typefaces across the five flavor associations in the exploratory experiment.

As shown in Figure 3, Typeface 5 (Yuan, bold strokes) had the highest selection rate for sweetness (57.38%) and was considered most representative of sweetness. On the spicy flavor dimension, Typeface 1 (Song, bold strokes) had the strongest association, with a selection rate of 65.57%. The bitter taste was mainly linked to Typefaces 7 (Calligraphic, with bifeng) and 1 (Song, bold strokes), with selection rates of 40.98% and 39.34%, respectively. For the salty taste, Typefaces 3 (Hei, without bifeng, bold strokes) and 1 (Song, with bifeng, bold strokes) had selection rates of 52.46% and 50.82%, respectively. For sour taste, Typeface 2 (Song, with bifeng, thin strokes) and Typeface 3 (Hei, without bifeng, bold strokes) were chosen at rates of 40.98% and 39.34%.

The results show that each typeface’s associative selections were not uniform across taste dimensions; they showed clear patterns (see Figure 3). Differences emerged, with clustering and consistent taste-association judgments. The rounded typeface (Yuan) had a higher selection proportion on the sweet dimension. Song and Calligraphic typefaces received more selections for sour, bitter, and spicy flavors. The Hei typeface was chosen more often for the salty dimension.

### 2.3. Discussion

The results of the exploratory experiment showed that, without any real taste stimuli, participants were able to complete taste association tasks based solely on the visual features of Chinese typefaces presented on real packaging, and the distribution differences were evident. As shown in Table 2 and Figure 3, in Category 1 (Song) and Category 2 (Hei), bold strokes generally had higher selection proportions than thin strokes across most flavors, such as spicy, bitter, and salty, indicating that stroke thickness affects taste associations. In Category 3 (Yuan), the purely rounded typeface (Typeface 5) had the highest selection rate for sweetness, while the typeface with mixed square-round features (Typeface 6) showed a marked decrease in sweet proportion, suggesting that inconsistency in roundness leads to differences in associations. In Category 4 (Calligraphic), the typeface with bifeng (Typeface 7) showed significantly higher association levels for bitter and spicy flavors than the typeface without bifeng (Typeface 8). Although this exploratory experiment did not include an independent pre-test, the data indicate that, with product expectations controlled, the typefaces themselves still possess substantial room for modulating taste perception. However, whether this modulatory mechanism is independent or interactive remains to be investigated. Based on the above findings, subsequent experiments will control variables by selecting the typefaces with higher selection proportions (Typefaces 1, 3, 5, 7) as representatives, systematically manipulating stroke thickness (bold, medium, thin) and the presence of bifeng, to further examine their interaction effects.

## 3. Main Study

### 3.1. Methods

#### 3.1.1. Design

The formal experiment adopted a 4 (font type) × 3 (stroke weight) within-subjects design. The typeface categories included Song, Hei, Yuan, and Calligraphic, corresponding to the representative samples numbered 1, 3, 5, and 7 in the exploratory experiment, respectively. Stroke thickness was divided into three levels: bold, medium, and thin. The dependent variable was participants’ ratings of each stimulus on the five flavor dimensions (sour, sweet, bitter, spicy, salty) using a 10-point Likert scale. According to the classification in Table 2, the Song and Calligraphic typefaces are those with bifeng, whereas the Hei and Yuan typefaces are those without bifeng.

The experiment retained the product packaging context from the exploratory experiment; only the typeface on the packaging was replaced to isolate the interference of other visual factors. Participants viewed five sets of stimulus materials in sequence (corresponding to the five flavors), each set containing 12 images (4 font type × 3 stroke weight), with the images presented in random order (Table 4).

#### 3.1.2. Participants

Participants in the study were required to have a Chinese cultural background to ensure the experimental stimuli could be effectively activated. Designers and participants who had previously taken part in the preliminary card sorting and the exploratory experiment were excluded from this experiment, thereby ensuring that all participants were exposed to the experimental stimuli for the first time. A total of 123 participants were recruited for this study. Following data cleaning, eight participants were excluded for not responding to at least one item or for providing identical responses for 8 consecutive items. In the end, 115 valid participants remained *(M_age_* = 35.73 ± 13.92). We conducted an a priori power analysis using G * Power 3.1.9.7 (effect size *f* = 0.10, *α* = 0.05, 1 − *β* = 0.85, *ε* = 0.75) based on a repeated-measures ANOVA (within-subjects design) framework to calculate the required sample size (Faul et al., 2007). The results indicated that the minimum required sample size was N = 86. This means that the sample size in the formal experiment clearly exceeded the required sample size. All valid data were included in subsequent analyses. Participant information is presented in Table 5.

#### 3.1.3. Procedure

The experiment used a combination of offline paper questionnaires and an online questionnaire platform (Wenjuanxing, https://www.wjx.cn/) for data collection. Participants first read an informed consent statement, confirming their voluntary participation and agreement to have their data used for academic research. Participants had the right to withdraw from the experiment at any time, in which case their data would not be included in the final analysis. This study used an anonymous questionnaire survey with minimal risk to participants. Voluntary completion and return of the questionnaire were taken to imply informed consent. Therefore, ethical approval was exempted, and the requirement for written informed consent was waived.

At the beginning of the experiment, participants were shown an overall image containing all 12 typeface images for a given taste set for 30 s. This phase aimed to familiarize participants with all typeface stimuli under that taste dimension and to establish an overall perceptual reference. After the overall image presentation, the rating phase began. The 12 typeface images were presented one by one, and participants rated each image according to the following instruction: Based on your first impression, please rate the strength of the association between the current image and the current taste. Ratings were made on a 10-point Likert scale, where 1 means ‘not at all’ and 10 means ‘extremely strong’.

Participants completed the five flavor sets (sour, sweet, bitter, spicy, and salty) independently (Table 4). Each set was conducted separately, and no feedback was provided between sets. Throughout the experiment, the experimenter did not interfere with participants’ judgments and was responsible only for recording evaluation results and participants’ verbal feedback (e.g., intuitive descriptions of typeface features). All data were collected anonymously and used solely for academic research. After the experiment, participants received a cash red envelope as a token of appreciation.

### 3.2. Results Analysis

This study employed linear mixed-effects models (LMMs) to examine the effects of Chinese typeface category and stroke thickness on crossmodal correspondences for the five flavors (sour, sweet, bitter, spicy, salty), following a similar experimental approach to that of Chuquichambi et al. (2024). Using SPSS 27, LMM analyses were conducted on the 12 stimulus combinations (4 typeface categories × 3 stroke thickness levels). In each model, typeface category (Song, Hei, Yuan, Calligraphic) and stroke thickness (bold, medium, thin) were entered as fixed effects, with a random-intercept-only structure (1|SubjectID). Given the within-subjects design of this study, the main source of non-independence lies in differences in individual baseline response tendencies—some participants consistently gave higher or lower taste expectancy ratings than others. The random intercept directly captures this characteristic, thereby effectively controlling for non-independence in the repeated-measures data. Matuschek et al. (2017) noted that, when selecting random-effect structures, adhering to the principle of parsimony can improve statistical power and avoid reduced power due to over-parameterization. All models were fitted using restricted maximum likelihood (REML), with degrees of freedom approximated using the Satterthwaite method. If the interaction between typeface category and stroke thickness was not significant, post hoc pairwise comparisons for main effects were conducted with Bonferroni correction (6 pairs for typeface category, 3 pairs for stroke thickness); if the interaction was significant, simple effects analyses were performed with appropriate corrections. Type III sums of squares were used for all fixed-effect tests.

Diagnostic checks were performed for each fitted model to assess model adequacy. Normality of residuals was evaluated using Q-Q plots of standardized residuals. For all five models, the Q-Q plots showed that the data points closely followed the diagonal line with no systematic deviations, indicating that the assumption of residual normality was met. Homoscedasticity was assessed by plotting standardized residuals against fitted values. The scatterplots showed that residuals were randomly distributed around the zero line, and the spread remained approximately constant across different ranges of fitted values, with no clear fan-shaped, funnel-shaped, or curved patterns, indicating that the assumption of homoscedasticity was satisfied. The intraclass correlation coefficient (ICC)—the proportion of total variance explained by the random intercept—was 0.283 for sour, 0.348 for sweet, 0.318 for bitter, 0.311 for spicy, and 0.287 for salty, indicating that approximately 28–35% of the total variance could be attributed to individual differences between participants, further supporting the necessity of including a random intercept. All models converged successfully without warning messages.

**Figure 4:**
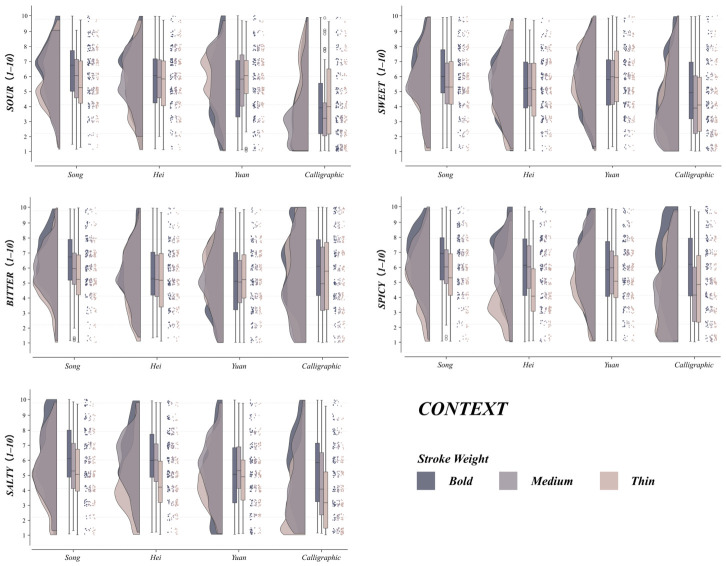
Raincloud plot of the effects of typeface category and stroke thickness on the five flavor expectations in the formal experiment. Dots represent data points from individual participants. A small number of outliers (shown as scatter points) reflect normal individual differences in the perception of typeface–flavor correspondences. These outliers were not removed in order to preserve the originality and representativeness of the data.

The results of the linear mixed-effects models (Figure 4) showed that typeface category had a significant effect on all five flavor expectations (Table 6). The effect was largest for sour taste, *F_(3, 1254)_* = 88.866, *p* < 0.001, partial *η*^2^ = 0.175 (large effect); followed by sweet taste, *F_(3, 1254)_* = 38.870, *p* < 0.001, partial *η*^2^ = 0.085 (medium effect); the effects on salty (partial *η*^2^ = 0.054, small effect), spicy (partial *η*^2^ = 0.038, small effect), and bitter (partial *η^2^* = 0.025, small effect) were relatively smaller but still significant. In contrast, the effects of stroke thickness showed a complementary pattern: they primarily acted on spicy (partial *η*^2^ = 0.081, medium effect) and salty (partial *η*^2^ = 0.062, medium effect), with no significant effect on sour (*p* = 0.136) and a negligible effect on sweet (partial *η*^2^ = 0.006). The interaction between typeface category and stroke thickness was significant for all five flavor dimensions (Table 6); therefore, further simple effects analyses were conducted to decompose the interactions. Table 7 presents the mean ratings of the five flavor expectations under different combinations of typeface category and stroke thickness.

#### 3.2.1. Simple Effects Analysis for Sour Taste Expectancy

The simple effects analysis for sour taste expectancy (Table 8) showed that the effect of stroke thickness was only present in Song and Calligraphic typefaces: for Song, the rating for bold stroke was significantly higher than that for thin stroke (*MD* = 0.672, *p* = 0.041); for Calligraphic, the rating for thin stroke was significantly higher than that for medium stroke (*MD* = 0.790, *p* = 0.002). When stroke thickness was controlled, across all typeface comparisons, the sour taste expectancy for the Calligraphic typeface was significantly lower than that for Song, Hei, and Yuan typefaces (*MDs* = 1.294–2.588, all *p* < 0.001). No other comparisons were significant.

#### 3.2.2. Simple Effects Analysis for Sweet Taste Expectancy

The simple effects analysis for sweet taste expectancy (Table 9) showed that the effect of stroke thickness was only present in the Calligraphic typeface: for Calligraphic, the rating for bold stroke was significantly higher than that for medium stroke (*MD* = 0.848, *p* = 0.001) and for thin stroke (*MD* = 0.756, *p* = 0.004). When stroke thickness was controlled, in most typeface comparisons, the sweet taste expectancy for the Calligraphic typeface was significantly lower than that for Song, Hei, and Yuan typefaces (*MDs* = 0.639–1.681, all *p* ≤ 0.039). In addition, a specific pattern emerged under the thin stroke condition: the rating for Yuan was significantly higher than that for Hei (*MD* = 0.933, *p* = 0.003). No other comparisons reached statistical significance.

#### 3.2.3. Simple Effects Analysis for Bitter Taste Expectancy

The simple effects analysis for bitter taste expectancy (Table 10) showed that the effect of stroke thickness was significant in both Song and Calligraphic typefaces: for Song, the rating for bold stroke was significantly higher than that for medium stroke (*MD* = 0.706, *p* = 0.028) and for thin stroke (*MD* = 1.042, *p* < 0.001); for Calligraphic, the rating for bold stroke was significantly higher than that for medium stroke (*MD* = 1.043, *p* < 0.001) and for thin stroke (*MD* = 0.664, *p* = 0.018). When stroke thickness was fixed: under the bold stroke condition, the rating for Song was significantly higher than that for Hei (*MD* = 0.874, *p* = 0.002) and for Yuan (*MD* = 1.429, *p* < 0.001), while the rating for Yuan was significantly lower than that for Calligraphic (*MD* = −1.026, *p* < 0.001); under the medium stroke condition, the rating for Song was significantly higher than that for Yuan (*MD* = 0.798, *p* = 0.010) and for Calligraphic (*MD* = 0.740, *p* = 0.036). No other comparisons reached statistical significance.

#### 3.2.4. Simple Effects Analysis for Spicy Flavor Expectancy

The simple effects analysis for spicy flavor expectancy (Table 11) showed that the effect of stroke thickness was significant within all four typefaces: for Song, the rating for bold stroke was significantly higher than that for medium stroke (*MD* = 0.605, *p* = 0.030) and for thin stroke (*MD* = 1.336, *p* < 0.001), and the rating for medium stroke was significantly higher than that for thin stroke (*MD* = 0.731, *p* = 0.018); for Hei, both bold and medium strokes were significantly higher than thin stroke (*MD* = 1.689, 1.437, both *p* < 0.001); for Yuan, both bold and medium strokes were significantly higher than thin stroke (*MD* = 0.572, *p* = 0.040; *MD* = 0.622, *p* = 0.030); for Calligraphic, the rating for bold stroke was significantly higher than that for medium stroke (*MD* = 1.698, *p* < 0.001) and for thin stroke (*MD* = 1.580, *p* < 0.001).

When stroke thickness was fixed: under the bold stroke condition, the rating for Song was significantly higher than that for Yuan (*MD* = 0.832, *p* = 0.003); under the medium stroke condition, the ratings for Song, Hei, and Yuan were significantly higher than that for Calligraphic (*MDs* = 1.673, 1.454, 1.496, all *p* < 0.001); under the thin stroke condition, the rating for Song was significantly higher than that for Hei (*MD* = 0.925, *p* = 0.001) and for Calligraphic (*MD* = 0.824, *p* = 0.010), and the rating for Yuan was significantly higher than that for Hei (*MD* = 0.857, *p* = 0.008). No other comparisons reached statistical significance.

#### 3.2.5. Simple Effects Analysis for Salty Taste Expectancy

The simple effects analysis for salty taste expectancy (Table 12) showed that the effect of stroke thickness was significant within Song, Hei, and Calligraphic typefaces: for Song, the rating for bold stroke was significantly higher than that for medium stroke (*MD* = 0.689, *p* = 0.023) and for thin stroke (*MD* = 1.143, *p* < 0.001); for Hei, both bold and medium strokes were significantly higher than thin stroke (*MD* = 1.345, 1.151, both *p* < 0.001); for Calligraphic, bold stroke was significantly higher than medium stroke (*MD* = 1.017, *p* < 0.001) and thin stroke (*MD* = 1.908, *p* < 0.001), and medium stroke was significantly higher than thin stroke (*MD* = 0.891, *p* = 0.003).

When stroke thickness was fixed: under the bold stroke condition, the rating for Song was significantly higher than that for Yuan (*MD* = 1.278, *p* < 0.001) and for Calligraphic (*MD* = 0.664, *p* = 0.016), and the rating for Hei was significantly higher than that for Yuan (*MD* = 0.984, *p* < 0.001); under the medium stroke condition, the ratings for Song, Hei, and Yuan were significantly higher than that for Calligraphic (*MDs* = 0.992, 1.193, 0.723, all *p* ≤ 0.004); under the thin stroke condition, the ratings for Song, Hei, and Yuan were also significantly higher than that for Calligraphic (*MDs* = 1.429, 0.933, 1.194, all *p* ≤ 0.001). No other comparisons reached statistical significance.

## 4. Discussion

Through an exploratory experiment and a formal experiment within a real packaging context, this study systematically investigated the interactive modulation effects of Chinese typeface categories (Song, Hei, Yuan, Calligraphic) and stroke thickness (bold, medium, thin) on the expectancy of the five flavors: sour, sweet, bitter, spicy, and salty. The results of linear mixed-effects models (LMMs) showed that the interaction effect between typeface category and stroke thickness was significant across all five flavor dimensions. Given the significant interaction effects, the main effects of typeface category and stroke thickness cannot independently explain changes in taste expectancy—their effects are interdependent. This paper analyzes the specific patterns of influence of typeface category and stroke thickness, respectively, across different taste dimensions, and further integrates their interaction mechanisms.

### 4.1. The Taste-Modulating Effect of Typeface Category

#### 4.1.1. The Sweetness Advantage of Yuan Typeface and Its Condition Dependence

The Yuan typeface had the highest mean rating on the sweet dimension, especially under the thin-stroke condition (*M* = 5.908). However, the simple effects analysis revealed that the differences in sweet ratings between bold, medium, and thin strokes within the Yuan typeface were not statistically significant (all *p* > 0.05). That is, although the numerical value for the thin stroke was higher than that for the bold stroke, it cannot yet be concluded that the bold stroke significantly weakens the perception of sweetness. Nevertheless, under the thin stroke condition, the sweet rating for Yuan was significantly higher than that for Hei (*MD* = 0.933, *p* = 0.003), and was numerically the highest among all typefaces; under the bold stroke condition, the differences between Yuan and Song or Hei were no longer significant. This finding suggests that the sweetness advantage of the Yuan typeface may be more pronounced when visual weight is lower (thin strokes), but the strength of this effect still requires further verification. Furthermore, on the sour dimension, the rating for Yuan under the thin-stroke condition (*M* = 5.756) was higher than under the bold-stroke condition (*M* = 5.210), but did not reach statistical significance, showing only a numerical reverse trend.

#### 4.1.2. The Association of Song Typeface’s Angular Bifeng with Bitter, Spicy, and Salty Tastes

The Song typeface under the bold stroke condition showed high ratings for bitter, spicy flavor, and salty taste expectancy (bitter: *M* = 6.538, spicy flavor: *M* = 6.639, salty: *M* = 6.118), all being the highest or second-highest among all typeface-thickness combinations. Simple effects analysis indicated that for Song, the bitter rating for bold stroke was significantly higher than that for medium stroke (*MD* = 0.706, *p* = 0.028) and thin stroke (*MD* = 1.042, *p* < 0.001); the spicy flavor rating was also significantly higher than for medium and thin strokes; for salty taste, bold stroke was significantly higher than thin stroke (*MD* = 1.143, *p* < 0.001). Moreover, under the bold stroke condition, the bitter rating for Song was significantly higher than that for Hei (*MD* = 0.874, *p* = 0.002) and for Yuan (*MD* = 1.429, *p* < 0.001); the spicy flavor rating was significantly higher than that for Yuan (*MD* = 0.832, *p* = 0.003). The experimental data also indicate that although the advantage of bold stroke over thin stroke for sour taste in Song was significant (*MD* = 0.672, *p* = 0.041), the absolute magnitude of this difference was smaller than the corresponding differences for bitter and spicy flavor, and there was no significant difference between medium and thin strokes, suggesting that the moderating effect of stroke thickness on sour taste may be less stable than for bitter and spicy flavor.

#### 4.1.3. The Hei Typeface’s Association with Saltiness and Glyph Compensation

The Hei typeface showed relatively high ratings on the salty dimension; under the bold stroke condition (*M* = 5.824), there was no significant difference from the Song typeface bold stroke (*M* = 6.118). For bitter and spicy flavor, the ratings for the Hei typeface bold stroke were significantly higher than those for its thin stroke (bitter: *MD* = 1.345, *p* < 0.001; spicy flavor: *MD* = 1.689, *p* < 0.001). However, the bitter rating for the Hei typeface bold stroke was significantly lower than that for the Song typeface bold stroke (*MD* = 0.874, *p* = 0.002), while there was no significant difference in spicy flavor between the Hei typeface and the Song typeface. This suggests that for the Hei typeface, a typeface without bifeng but square and rigid, the enhancing effect of visual weight (bold stroke) on bitter, spicy flavor, and salty also exists, but bitter may require more explicit angular bifeng features (such as those in the Song typeface) to reach its highest level.

#### 4.1.4. The Dynamic Bifeng of Calligraphic Typeface and the Effect of Visual Complexity

The Calligraphic typeface showed a rating pattern across multiple taste dimensions that differed from those of other typefaces, with its effects unstable and heavily influenced by stroke thickness.

On the bitter and spicy flavor dimensions, the Calligraphic typeface exhibited high ratings for bitter (*M* = 6.135) and spicy flavor (*M* = 6.059) only under the bold stroke condition, approaching the level of Song bold stroke; however, ratings dropped sharply under the medium stroke condition (bitter: *M* = 5.092, spicy flavor: *M* = 4.361), and increased slightly under the thin stroke condition (bitter: *M* = 5.471, spicy flavor: *M* = 4.479). Simple effects analysis confirmed that for Calligraphic, the bitter rating for bold stroke was significantly higher than that for medium stroke (*MD* = 1.043, *p* < 0.001) and thin stroke (*MD* = 0.664, *p* = 0.018); the spicy flavor rating was also significantly higher than for medium and thin strokes (both *p* < 0.001). However, there was no significant difference between medium and thin strokes for bitter (MD = 0.379, *p* > 0.05), nor for spicy flavor (*MD* = 0.118, *p* > 0.05). This discontinuous pattern contrasts with the gradual pattern observed for the Song typeface.

On the sweet dimension, regardless of stroke thickness, the sweet ratings for the Calligraphic typeface were the lowest among the four typefaces, and were significantly lower than those for Yuan under bold, medium, and thin stroke conditions (all *p* ≤ 0.039). On the salty dimension, the salty ratings for the Calligraphic typeface fluctuated dramatically with stroke thickness: they were relatively high under the bold stroke condition (*M* = 5.454), then decreased successively under medium (*M* = 4.437) and thin (*M* = 3.546) strokes, with all pairwise comparisons being significant (Table 12). On the sour dimension, the rating for Calligraphic under the thin stroke condition (*M* = 4.168) was significantly higher than under the medium stroke condition (*MD* = 0.790, *p* = 0.002), with the bold stroke condition falling between. This ‘V-shaped’ pattern suggests that the sour association of the Calligraphic typeface may not depend on visual weight; instead, it is stronger under thin strokes (where the overall appearance is leaner and sharpness is enhanced).

Taken together, the gustatory crossmodal correspondences of the Calligraphic typeface cannot be simply subsumed into a linear framework of ‘with bifeng vs. without bifeng’ or ‘bold vs. thin’. Its dynamic bifeng, highly complex visual features, and cultural symbolic meanings together produce an unstable and directionally diverse pattern of interactions.

### 4.2. The Condition Dependence of Stroke Thickness

The effect of stroke thickness showed clear typeface dependence across different taste dimensions. Overall, bold strokes elicited higher ratings than thin strokes on the bitter, spicy, and salty dimensions, but this effect did not hold across all typefaces. For example, in the Song and Hei typefaces, the ratings for bitter, spicy flavor, and salty under bold strokes were significantly higher than those under thin strokes; however, in the Yuan typeface, most differences between bold and thin strokes on bitter, spicy flavor, and salty were not significant (except for spicy flavor: bold vs. thin *MD* = 0.572, *p* = 0.040). This suggests that the moderating effect of stroke thickness on taste intensity occurs mainly in typefaces with angular bifeng or square features, whereas for rounded typefaces, the modulation of taste intensity by stroke thickness is very limited.

On the sour and sweet dimensions, the effect of stroke thickness was even weaker and varied in direction. The dominant factor for sweet taste is glyph curvature rather than thickness, while sour taste showed a typeface-dependent thickness effect (bold stroke enhanced sourness in the Song typeface, while the Yuan typeface showed numerically higher ratings under thin strokes, though not significant). This pattern further supports the core idea of the interactive modulation model: visual weight does not map independently onto taste intensity but must operate synergistically with the typeface’s contour features.

### 4.3. Theoretical Integration of Interaction Patterns

The experimental results align with the previous hypotheses: there exists a non-linear synergistic or inhibitory relationship between typeface category and stroke thickness, jointly shaping taste expectancy. Three typical interaction patterns between typeface category and stroke thickness can be summarised:

Synergistic enhancement type (observed for bitter, spicy flavor, and salty). When angular bifeng or square typefaces (Song, Hei) are combined with bold strokes, taste expectancy is significantly amplified.

Condition-dependent type (observed for sweet taste). The rounded typeface (Yuan) showed a sweetness advantage only under the thin stroke condition.

Dual-path reverse modulation type (observed for sour taste). The angular bifeng typeface (Song) followed the rule of bold-stroke enhancement, whereas the rounded typeface (Yuan) showed a reverse trend with numerically higher ratings under thin strokes. One possible explanation for this divergent pattern is that sour taste associations may have dual semantic attributes: on the one hand, a warning signal of unripe fruit (associated with angularity and intensity); on the other hand, a refreshing and bright positive attribute (associated with slenderness and sharpness). However, this interpretation is currently only a preliminary account and awaits further validation.

### 4.4. Commonalities and Differences in Typeface–Flavor Crossmodal Correspondences Between Chinese and Western Contexts

Crossmodal correspondence research on typeface–taste associations in Chinese and Western contexts reveals both cross-culturally consistent perceptual regularities and differences arising from writing systems, visual features, and cultural experience. Studies in both contexts share the cross-cultural core regularity of ‘curvature-sweetness, angularity-non-sweetness’, and both indicate that visual features interact synergistically rather than simply additively. At the same time, the feature-driven pathway of the Chinese character system, the dual geometric and cultural attributes of bifeng, and the high visual complexity of the Calligraphic typeface together shape the specific patterns of taste associations in the Chinese context.

#### 4.4.1. Commonalities: Cross-Cultural Perceptual Regularities

Crossmodal correspondence research on typeface–taste associations in Chinese and Western contexts reveals consistent underlying regularities. In the present study, the Yuan typeface under the thin stroke condition showed the highest sweet taste expectancy, which aligns with the ‘rounded-sweet’ correspondence found in Latin-script studies (Velasco et al., 2015b, 2018). Meanwhile, the high ratings of the Song typeface with bold strokes for non-sweet tastes such as bitter, spicy flavor, and salty are also consistent with the ‘angular-bitter/sour’ mapping observed in Western research. This cross-cultural consistency suggests that ‘curvature-sweetness, angularity-non-sweetness’ may constitute a universal crossmodal mapping rule, underpinned by a dual mechanism of affective mediation (Salgado-Montejo et al., 2015) and statistical learning (Spence, 2011). Furthermore, in the Chinese character system, the effects of typeface category and stroke thickness (including bifeng) on flavor expectancy are not independent and additive but operate together in a non-linear manner. This is consistent with the phenomenon of ‘synergistic combination of visual features’ highlighted in Western research (Lee & Spence, 2023).

#### 4.4.2. Differences: Modulation of Gustatory Crossmodal Correspondence Pathways by Writing Systems

In terms of perceptual pathways, Chinese characters tend to follow a ‘feature-driven’ route. As logograms, the discrete structure of Chinese characters makes it easy for observers to extract visual information from local features such as bifeng morphology, turning sharpness, and stroke thickness, and to activate taste associations accordingly. Tavassoli (2001) found that Chinese character users had significantly better memory for character colours than alphabet users, indicating a higher degree of visual engagement in Chinese character processing. In the present study, stroke thickness significantly modulated the expectancy of intense tastes such as spicy flavor, salty, and bitter, and this effect depended on the typeface category. Specifically, the modulation of bitter taste expectancy by stroke thickness was not universally effective across all typefaces but was significant only for the Song typeface with its angular bifeng, while it had almost no effect on the rounded, bifeng-free Yuan typeface. This reflects the driving role of local features (bifeng, turning sharpness) in gustatory crossmodal correspondences within the Chinese character system. In contrast, alphabetic scripts tend to follow a more ‘holistic-driven’ route, where taste associations rely more on the overall contours and curvatures of letters or words.

Regarding the dual geometric and cultural attributes of bifeng, the Chinese character system exhibits a unique pattern. A quantitative study by Lyu et al. (2022) found that in the aesthetic evaluation of regular script (Kai), bifeng had a much stronger influence on style perception than did structure. The angular bifeng is a core visual feature of the Song typeface. The simple effects analysis of the present study shows that the high ratings of the Song typeface for bitter, spicy flavor, and salty did not arise solely from the presence of bifeng, but from the synergistic amplification of ‘bold stroke + angular bifeng’. Under the bold stroke condition, the bitter rating for Song was significantly higher than that for Hei and Yuan, but this difference diminished or disappeared under medium and thin stroke conditions. This suggests that angular bifeng does not, by itself, automatically convey strong taste expectations; rather, it requires sufficient visual weight (bold strokes) to produce a significant enhancement. Moreover, a study by Wei et al. (2018) showed that the visual-spatial features of Chinese characters (upright vs. slanted) can systematically activate different semantic associations (reliability vs. innovation), indicating that local visual features of Chinese characters can activate specific semantic meanings through embodied pathways. By analogy, the angularity of bifeng may similarly activate semantic associations related to intense tastes through embodied pathways. Chinese bifeng not only carries geometric features but also embodies traditional Chinese bodily based concepts and cultural philosophy, such as ‘力道’ (forcefulness), ‘筋骨’ (muscularity), ‘势’ (dynamic potential), and ‘天人合一’ (harmony between heaven and humanity). These cultural meanings further reinforce the association between angular bifeng and intense tastes (especially spicy flavor and bitter), creating a hybrid pathway that combines visual features with cultural experience. In contrast to the standardised presentation of bifeng in printed typefaces such as Song, calligraphic typefaces have more complex stroke-terminal morphology, more stroke intersections, and more complex overall contours (Y. Wang & Lyu, 2024), exhibiting greater formal variability and richer connotations, and consequently more complex flavour crossmodal correspondences. For example, the instability of taste expectancy for the Calligraphic typeface under medium stroke thickness may arise from such visual heterogeneity, where multiple features compete simultaneously for processing resources, failing to form a dominant dimension that drives a specific taste association. However, this interpretation still requires further experimental validation, for example, by systematically manipulating the degree of bifeng consistency to test its direct effect on taste expectancy.

Finally, cultural experience modulates taste mappings. The present study included spicy flavor as an independent dimension, and the results showed that bold strokes combined with angular bifeng (especially in the Song typeface) enhanced spicy flavor expectancy, whereas thin strokes weakened it. Physiologically, spiciness is not a taste category but a compound of pain and taste, yet it holds a high perceptual status in Chinese food culture and shows significant crossmodal correspondences.

### 4.5. Methodological Implications of the Real Packaging Context

This study employed real food packaging as stimulus materials, differing from previous experimental paradigms that used abstract shapes or isolated typefaces. Liu et al. (2020) classified packaging elements into linguistic and non-linguistic elements, noting that they influence consumers through different mechanisms—non-linguistic elements primarily affect perception and affective preferences, while linguistic elements primarily influence behavioral responses, and both are modulated by multisensory interaction mechanisms. Tu and Yang (2013) investigated the mechanisms through which packaging influences food taste experience, emphasizing the critical role of multisensory integration in food packaging. Peng and Wan (2022) examined crossmodal influences of taste stimuli on visual attention in Chinese participants, finding that flavor cues can induce attentional bias toward associated colours, indicating that stable taste–visual crossmodal associations exist among Chinese consumers. By presenting typefaces on real packaging, the present study not only improved ecological validity but also verified the transferability of typeface–taste crossmodal effects to real consumption contexts. However, real packaging inevitably introduces additional variables such as colour, material, and brand information. This is both an advantage in terms of ecological validity and a methodological limitation that needs to be acknowledged.

### 4.6. Theoretical Contributions and Practical Implications

The theoretical contributions of this study are threefold. First, it extends crossmodal correspondence research from Western alphabetic writing systems to the logographic Chinese character system, validating the cross-script universality of the synergistic combination effect of visual features. Second, it proposes a synergistic framework centred on interaction effects, replacing the traditional main-effect framework, and emphasises that the non-linear combination of typeface category and stroke thickness determines the specific direction of taste expectancy. Third, it documents differential modulatory patterns of typeface categories on flavour perception, as well as the atypical sensitivity of the Calligraphic typeface across multiple taste dimensions. These findings demonstrate that the complexity of crossmodal mappings far exceeds simple linear correspondences, although the detailed cognitive mechanisms remain to be further empirically validated.

At the practical level, this study provides designers and marketers with an evidence-based reference for typeface–flavour configurations. Perceptual congruity theory suggests that the degree of match between typeface features and product attributes significantly influences consumer evaluations (Doyle & Bottomley, 2004, 2006). The present study further indicates that such congruity requires simultaneous consideration of the interaction between typeface category and stroke thickness, rather than a single dimension. Sweetness-oriented brands (e.g., desserts, milk tea) should adopt rounded typefaces combined with thin strokes to form a consistent ‘sweet’ visual language in brand logos and packaging. Bitterness- or spiciness-oriented brands (e.g., dark chocolate, spicy snacks) should adopt typefaces with angular bifeng combined with bold strokes to convey strong taste cues. Salty products may consider typefaces with angular bifeng or square features combined with bold strokes. For sour products, the choice should be flexible, aligned with specific brand positioning: angular bifeng typefaces tend toward bold strokes, while rounded typefaces tend toward thin strokes. Moreover, the Calligraphic typeface fully activates the expectancy of bitter and spicy flavour only under the bold stroke condition, while its taste expectancy is unstable under medium strokes. Therefore, the application of the Calligraphic typeface requires careful matching of stroke thickness with the target taste positioning.

### 4.7. Limitations and Future Directions

First, this study operationalised bifeng as a binary variable (present vs. absent) rather than as a continuously measurable geometric variable. The bifeng of the Song typeface takes the form of regular, geometric decorative terminals, whereas the bifeng of the Calligraphic typeface exhibits irregular, organic variations. These two types differ substantially along continuous dimensions such as sharpness, angle, and curvature, and collapsing them into a binary classification inevitably loses important perceptual information. However, this operational choice should be understood within the developmental trajectory of crossmodal correspondence research: Western shape–taste research similarly evolved from a binary classification of ‘angular vs. rounded’ to continuous curvature measurements (Velasco et al., 2014, 2015b, 2016a); the binary operationalisation of bifeng in this study lies at a similar preliminary exploratory stage and has empirical value in establishing whether bifeng participates in gustatory crossmodal correspondences. Furthermore, bifeng and Western serifs may differ in morphological variability, cultural semantic load (Zhao et al., 2017, 2018), and visual salience, but the above analysis lacks independent perceptual validation. Future research could draw on geometric quantification methods for stroke terminals (X. Wang et al., 2013), adopt parametric approaches to independently manipulate the sharpness, length, and curvature of bifeng, further test the net effect of bifeng on taste expectancy, and conduct comparative studies between the two.

Second, although we introduced real packaging designs to enhance ecological validity, this inevitably introduced additional variables. Beyond colour, material, and brand information, the products themselves carry strong inherent flavour expectations, which may have interacted with the typeface effects to influence participants’ taste ratings. This study did not conduct an independent pre-test to quantify each product’s baseline flavour expectancy, nor did it assess whether packaging familiarity dominated participants’ judgments. Consequently, we cannot fully attribute the observed rating differences solely to typeface features; it remains unclear to what extent typeface effects reflect independent crossmodal associations rather than modulation of pre-existing product expectations. In future research, we will first establish mapping models through tightly controlled basic experiments (using abstract stimuli without product context), then gradually increase contextual realism, and directly compare typeface effects with and without packaging to isolate the net contribution of typeface features. At the same time, in advertising and social media communication, typeface combinations can be integrated with other visual elements such as colour, to create multidimensional sensory congruity, reinforcing consumers’ expectations of product flavour characteristics. However, this study measured only taste expectancy; the actual effects of typeface selection on brand emotional attachment, cultural identity, or purchasing behaviour await further empirical validation.

Finally, the taste dimensions explored in this study are primarily limited to sour, sweet, bitter, spicy, and salty, and have not yet included umami. As a widely recognised fifth basic taste, umami plays an important role in food perception. Whether stable taste mappings exist between umami and the visual features of Chinese typefaces remains to be verified in future research.

## 5. Conclusions

This study confirms that the visual features of Chinese typefaces play an important role in shaping taste expectancy. It not only reaffirms the cross-cultural perceptual rule that ‘roundness is associated with sweetness, angularity with non-sweet tastes’, but further reveals that in the unique logographic writing system of Chinese characters, typeface category, stroke thickness, and the presence of bifeng can all serve as critical visual cues influencing taste perception.

These findings extend the application of multisensory integration and crossmodal correspondence theories to a non-alphabetic writing system, and provide empirical evidence for sensory marketing and visual communication design. In practical application, typeface selection should not be viewed merely as a stylistic choice, but should be understood as a precise perceptual language. By scientifically configuring the visual attributes of typefaces, designers can construct a guiding taste narrative for consumers even before the product is experienced.

## Figures and Tables

**Table 1:** Font Samples.

Calligraphic Fonts	Classical-Style Fonts	Modern Fonts	Creative Fonts	Artistic Fonts
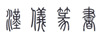	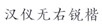	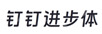	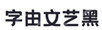	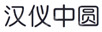
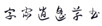	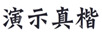	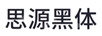	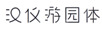	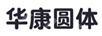
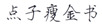	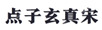	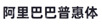	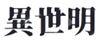	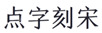
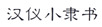	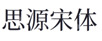	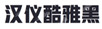	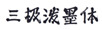	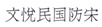

**Table 2:** Font analysis in the exploratory experiment.

Grouping	ID	Characteristics		Font Legend	Font Type
		Contact	Distinction		
Category 1	1	Distinct bifeng, pronounced stroke width variation, upright	Bold	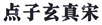	Song
	2		Thin	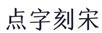	
Category 2	3	Indistinct bifeng, uniform stroke width, upright	Bold	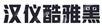	Hei
	4		Thin	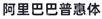	
Category 3	5	Indistinct bifeng, uniform stroke width, rounded	Rounded	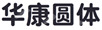	Yuan
	6		Square and Round	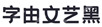	
Category 4	7	Graphic symbolization of strokes	Distinct bifeng	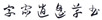	Calligraphic
	8		Indistinct bifeng	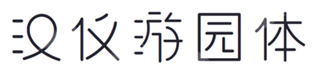	

**Table 3:** Basic information of participants in the exploratory experiment (N = 80).

Demographics	Classification	Frequency	Percent (%)
Gender	Male	39	48.75
	Female	41	51.25
Age	0–17	8	10.00
	18–44	49	61.25
	45–59	18	22.50
	60+	5	6.25

**Table 4:** Full factorial combination of font type (Song, Hei, Yuan, Calligraphic) and stroke weight (bold, medium, thin) with corresponding packaging stimuli for each of the five flavor categories (sour, sweet, bitter, spicy, salty).

Font Type	Thickness	ID	Experimental Sample				
			Sour	Sweet	Bitter	Spicy	Salty
Song	Bold	1	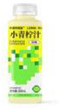	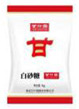	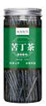	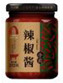	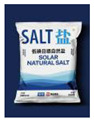
	Medium	2	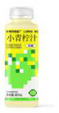	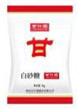	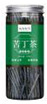	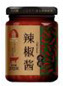	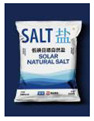
	Thin	3	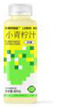	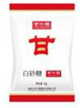	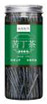	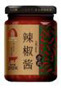	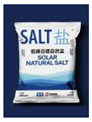
Hei	Bold	4	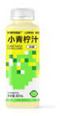	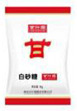	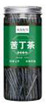	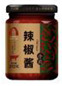	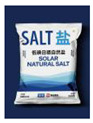
	Medium	5	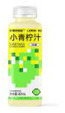	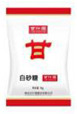	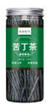	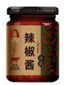	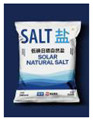
	Thin	6	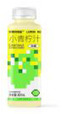	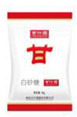	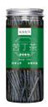	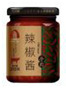	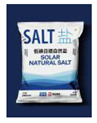
Yuan	Bold	7	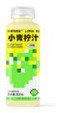	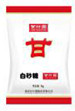	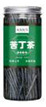	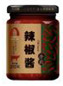	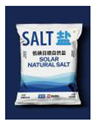
	Medium	8	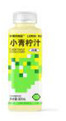	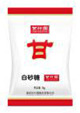	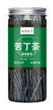	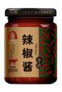	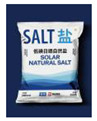
	Thin	9	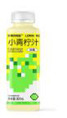	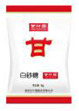	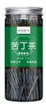	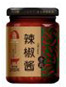	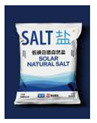
Calligraphic	Bold	10	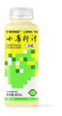	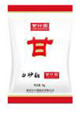	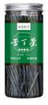	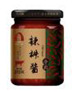	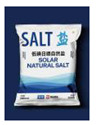
	Medium	11	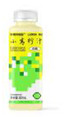	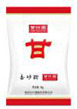	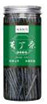	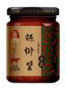	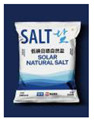
	Thin	12	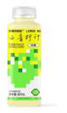	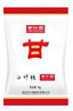	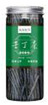	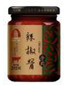	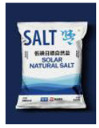

**Table 5:** Basic information of participants in the formal experiment (N = 115).

Demographics	Classification	Frequency	Percent (%)
Gender	Male	48	41.74
	Female	67	58.26
Age	0–17	13	11.30
	18–44	66	57.39
	45–59	29	25.22
	60+	7	6.09

**Table 6:** LMM fixed-effects tests for the effects of typeface category, stroke thickness, and their interaction on flavor expectations.

		Sour	Sweet	Bitter	Spicy	Salty
Font type	*F*	88.886	38.870	10.734	16.416	22.738
	*p*	<0.001 ***	<0.001 ***	<0.001 ***	<0.001 ***	<0.001 ***
	partial *η*^2^	0.175	0.085	0.025	0.038	0.054
Stroke weight	*F*	1.998	4.094	9.850	55.278	41.149
	*p*	0.136	0.017 *	<0.001 ***	<0.001 ***	<0.001 ***
	partial *η*^2^	0.003	0.006	0.015	0.081	0.062
Font type × Stroke weight	*F*	3.457	2.637	2.803	7.092	4.963
	*p*	0.002 **	0.015 *	0.010 *	<0.001 ***	<0.001 ***
	partial *η*^2^	0.016	0.012	0.013	0.033	0.023

Note. For all F-tests, the denominator degrees of freedom were approximated using the Satterthwaite method. Effect sizes are partial eta-squared (partial *η*^2^). Effect size magnitude interpretation follows Cohen (1988): small = 0.01, medium = 0.06, large = 0.14. * *p* < 0.05, ** *p* < 0.01, *** *p* < 0.001.

**Table 7:** Mean ratings of five-flavor expectancy across different typeface categories and stroke thickness levels.

Font Type	Stroke Weight	Sour		Sweet		Bitter		Spicy		Salty	
		*M*	*SD*	*M*	*SD*	*M*	*SD*	*M*	*SD*	*M*	*SD*
Song	Bold	6.361	2.082	5.992	2.287	6.538	1.956	6.639	1.986	6.118	2.565
	Medium	5.966	2.008	5.521	2.147	5.832	1.919	6.034	1.961	5.429	2.177
	Thin	5.689	2.057	5.496	2.201	5.496	1.987	5.303	2.106	4.975	2.279
Hei	Bold	5.882	2.241	5.244	2.091	5.664	2.108	6.067	2.250	5.824	2.389
	Medium	5.689	1.956	5.395	2.005	5.538	2.016	5.815	2.107	5.630	2.143
	Thin	5.605	2.293	4.975	2.234	5.16	2.198	4.378	2.107	4.479	2.251
Yuan	Bold	5.210	2.541	5.622	2.307	5.109	2.386	5.807	2.412	4.840	2.642
	Medium	5.479	2.299	5.765	2.212	5.034	2.251	5.857	2.226	5.160	2.285
	Thin	5.756	1.966	5.908	2.079	5.202	2.196	5.235	2.090	4.740	2.149
Calligraphic	Bold	3.916	2.53	4.983	2.531	6.135	2.548	6.059	2.65	5.454	2.583
	Medium	3.378	2.236	4.135	2.524	5.092	2.746	4.361	2.540	4.437	2.527
	Thin	4.168	2.611	4.227	2.309	5.471	2.776	4.479	2.597	3.546	2.280

**Table 8:** Pairwise comparisons within fixed effect levels for the simple effects analysis of sour taste expectancy.

Fixed Effects		Pairwise Comparisons	*MD*	*p*	95% *CI*
Font type	Song	Bold vs. Thin	0.672	0.041 *	[0.019, 1.199]
	Calligraphic	Thin vs. Medium	0.790	0.002 **	[0.236, 1.416]
Stroke weight	Bold	Song vs. Yuan	1.151	<0.001 ***	[0.384, 1.685]
		Song vs. Calligraphic	2.445	<0.001 ***	[1.671, 2.972]
		Hei vs. Calligraphic	1.966	<0.001 ***	[1.280, 2.581]
		Yuan vs. Calligraphic	1.294	<0.001 ***	[0.637, 1.937]
	Medium	Song vs. Calligraphic	2.588	<0.001 ***	[1.819, 3.120]
		Hei vs. Calligraphic	2.311	<0.001 ***	[1.619, 2.920]
		Yuan vs. Calligraphic	2.101	<0.001 ***	[1.454, 2.755]
	Thin	Song vs. Calligraphic	1.521	<0.001 ***	[0.776, 2.076]
		Hei vs. Calligraphic	1.437	<0.001 ***	[0.732, 2.033]
		Yuan vs. Calligraphic	1.588	<0.001 ***	[0.932, 2.233]

Note. * *p* < 0.05, ** *p* < 0.01, *** *p* < 0.001.

**Table 9:** Pairwise comparisons within fixed effect levels for the simple effects analysis of sweet taste expectancy.

Fixed Effects		Pairwise Comparisons	*MD*	*p*	95% *CI*
Font type	Calligraphic	Bold vs. Medium	0.848	0.001 **	[0.294, 1.427]
		Bold vs. Thin	0.756	0.004 **	[0.199, 1.332]
Stroke weight	Bold	Song vs. Hei	0.748	0.011 *	[0.115, 1.364]
		Song vs. Calligraphic	0.009	<0.001 ***	[0.393, 1.642]
		Yuan vs. Calligraphic	0.639	0.039 *	[0.019, 1.268]
	Medium	Song vs. Calligraphic	0.386	<0.001 ***	[0.845, 2.094]
		Hei vs. Calligraphic	0.260	<0.001 ***	[0.619, 1.868]
		Yuan vs. Calligraphic	0.630	<0.001 ***	[0.949, 2.198]
	Thin	Song vs. Calligraphic	0.269	<0.001 ***	[0.662, 1.912]
		Hei vs. Yuan	−0.933	0.003 **	[−1.451, −0.202]
		Hei vs. Calligraphic	0.748	<0.001 ***	[0.202, 1.451]
		Yuan vs. Calligraphic	1.681	<0.001 ***	[1.028, 2.277]

Note. * *p* < 0.05, ** *p* < 0.01, *** *p* < 0.001.

**Table 10:** Pairwise comparisons within fixed effect levels for the simple effects analysis of bitter taste expectancy.

Fixed Effects		Pairwise Comparisons	*MD*	*p*	95% *CI*
Font type	Song	Bold vs. Medium	0.706	0.028 *	[0.051, 1.218]
		Bold vs. Thin	1.042	<0.001 ***	[0.399, 1.566]
	Calligraphic	Bold vs. Medium	1.043	<0.001 ***	[0.365, 1.531]
		Bold vs. Thin	0.664	0.018 *	[0.086, 1.253]
Stroke weight	Bold	Song vs. Hei	0.874	0.002 **	[0.244, 1.530]
		Song vs. Yuan	1.429	<0.001 ***	[0.687, 1.973]
		Yuan vs. Calligraphic	−1.026	<0.001 ***	[−1.617, −0.331]
	Medium	Song vs. Yuan	0.798	0.010 *	[0.122, 1.408]
		Song vs. Calligraphic	0.740	0.036 *	[0.027, 1.313]

Note. * *p* < 0.05, ** *p* < 0.01, *** *p* < 0.001.

**Table 11:** Pairwise comparisons within fixed effect levels for the simple effects analysis of spicy flavor expectancy.

Fixed Effects		Pairwise Comparisons	*MD*	*p*	95% *CI*
Font type	Song	Bold vs. Medium	0.605	0.030 *	[0.046, 1.224]
		Bold vs. Thin	1.336	<0.001 ***	[0.724, 1.902]
		Medium vs. Thin	0.731	0.018 *	[0.089, 1.267]
	Hei	Bold vs. Thin	1.689	<0.001 ***	[1.124, 2.302]
		Medium vs. Thin	1.437	<0.001 ***	[0.907, 2.084]
	Yuan	Bold vs. Thin	0.572	0.040 *	[0.020, 1.198]
		Medium vs. Thin	0.622	0.030 *	[0.046, 1.224]
	Calligraphic	Bold vs. Medium	1.698	<0.001 ***	[1.116, 2.293]
		Bold vs. Thin	1.580	<0.001 ***	[0.942, 2.119]
Stroke weight	Bold	Song vs. Yuan	0.832	0.003 **	[0.212, 1.510]
	Medium	Song vs. Calligraphic	1.673	<0.001 ***	[0.977, 2.275]
		Hei vs. Calligraphic	1.454	<0.001 ***	[0.847, 2.145]
		Yuan vs. Calligraphic	1.496	<0.001 ***	[0.777, 2.075]
	Thin	Song vs. Hei	0.925	0.001 **	[0.299, 1.597]
		Song vs. Calligraphic	0.824	0.010 *	[0.125, 1.423]
		Hei vs. Yuan	−0.857	0.008 **	[−1.440, −0.142]

Note. * *p* < 0.05, ** *p* < 0.01, *** *p* < 0.001.

**Table 12:** Pairwise comparisons within fixed effect levels for the simple effects analysis of salty taste expectancy.

Fixed Effects		Pairwise Comparisons	*MD*	*p*	95% *CI*
Font type	Song	Bold vs. Medium	0.689	0.023 *	[0.070, 1.286]
		Bold vs. Thin	1.143	<0.001 ***	[0.497, 1.712]
	Hei	Bold vs. Thin	1.345	<0.001 ***	[0.784, 1.999]
		Medium vs. Thin	1.151	<0.001 ***	[0.575, 1.790]
	Calligraphic	Bold vs. Medium	1.017	<0.001 ***	[0.410, 1.625]
		Bold vs. Thin	1.908	<0.001 ***	[1.244, 2.460]
		Medium vs. Thin	0.891	0.003 **	[0.227, 1.443]
Stroke weight	Bold	Song vs. Yuan	1.278	<0.001 ***	[0.574, 1.913]
		Song vs. Calligraphic	0.664	0.016 *	[0.095, 1.435]
		Hei vs. Yuan	0.984	<0.001 ***	[0.347, 1.687]
	Medium	Song vs. Calligraphic	0.992	<0.001 ***	[0.434, 1.774]
		Hei vs. Calligraphic	1.193	<0.001 ***	[0.678, 2.018]
		Yuan vs. Calligraphic	0.723	0.004 **	[0.200, 1.540]
	Thin	Song vs. Calligraphic	1.429	<0.001 ***	[0.843, 2.183]
		Hei vs. Calligraphic	0.933	0.001 **	[0.330, 1.670]
		Yuan vs. Calligraphic	1.194	<0.001 ***	[0.521, 1.861]

Note. * *p* < 0.05, ** *p* < 0.01, *** *p* < 0.001.

## Data Availability

The authors confirm that the data supporting the findings of this study are available within the article.
